# Sumatran tiger survival threatened by deforestation despite increasing densities in parks

**DOI:** 10.1038/s41467-017-01656-4

**Published:** 2017-12-05

**Authors:** Matthew Scott Luskin, Wido Rizki Albert, Mathias W. Tobler

**Affiliations:** 1 Center for Tropical Forest Science – Forest Global Earth Observatory, Smithsonian Tropical Research Institute, PO Box 37012, Washington, DC 20012 USA; 2 Asian School of the Environment, Nanyang Technical University, 50 Nanyang Avenue, N2-01c-36, Singapore, 639798 Singapore; 3 Department of Environmental Science, Policy, and Management, University of California, Berkeley, 204 Mulford Hall, Berkeley, CA 94720 USA; 4 Fauna & Flora International - Indonesia Programme, PO Box 42, Sungai Penuh, Kerinci, Jambi, 37111 Sumatra Indonesia; 5 San Diego Zoo Global, Institute for Conservation Research, 15600 San Pasqual Valley Road, Escondido, CA 92027-7000 USA

## Abstract

The continuing development of improved capture–recapture (CR) modeling techniques used to study apex predators has also limited robust temporal and cross-site analyses due to different methods employed. We develop an approach to standardize older non-spatial CR and newer spatial CR density estimates and examine trends for critically endangered Sumatran tigers (*Panthera tigris sumatrae*) using a meta-regression of 17 existing densities and new estimates from our own fieldwork. We find that tiger densities were 47% higher in primary versus degraded forests and, unexpectedly, increased 4.9% per yr from 1996 to 2014, likely indicating a recovery from earlier poaching. However, while tiger numbers may have temporarily risen, the total potential island-wide population declined by 16.6% from 2000 to 2012 due to forest loss and degradation and subpopulations are significantly more fragmented. Thus, despite increasing densities in smaller parks, we conclude that there are only two robust populations left with >30 breeding females, indicating Sumatran tigers still face a high risk of extinction unless deforestation can be controlled.

## Abstract (web summary)

Determining the conservation status and populations trends for elusive predators has been impeded by discrepancies in how densities are calculated. Here, Luskin et al. introduce a means to standardize previous density estimates and assess the threats to the Sumatran tiger.

## Introduction

Robust assessments of the spatial distribution and population dynamics of threatened species are crucial for designing effective conservation policies^1^. This is often impeded by methodological differences employed by researchers to collect and analyze data^2, 3^. Obtaining information on rare apex predators is particularly difficult given their large home ranges, low population densities, and often cryptic nature^4–6^. The widespread proliferation of camera-trap (CT) surveys over the last 15 years, which provide an efficient means of monitoring abundance across wide geographic ranges, has partially solved this problem. Still, the use of CT data to compare trends across diverse sites remains controversial despite its potential to illuminate population dynamics far more than any single-site analysis^7–9^. An important source of controversy arises in how to deal with the known biases inherent in non-spatial capture–recapture (CR) methods, which are prone to overestimating densities by as much as 20–60%^5^. CR analyses have recently been supplanted by more robust spatial capture–recapture (SCR) approaches. Thus, accurate estimation of population trends for endangered apex predators requires a method that can integrate older CR estimates with more recent SCR estimates, whilst accounting for the biases in the former and variation in error associated with every estimate.

**Fig. 1:**
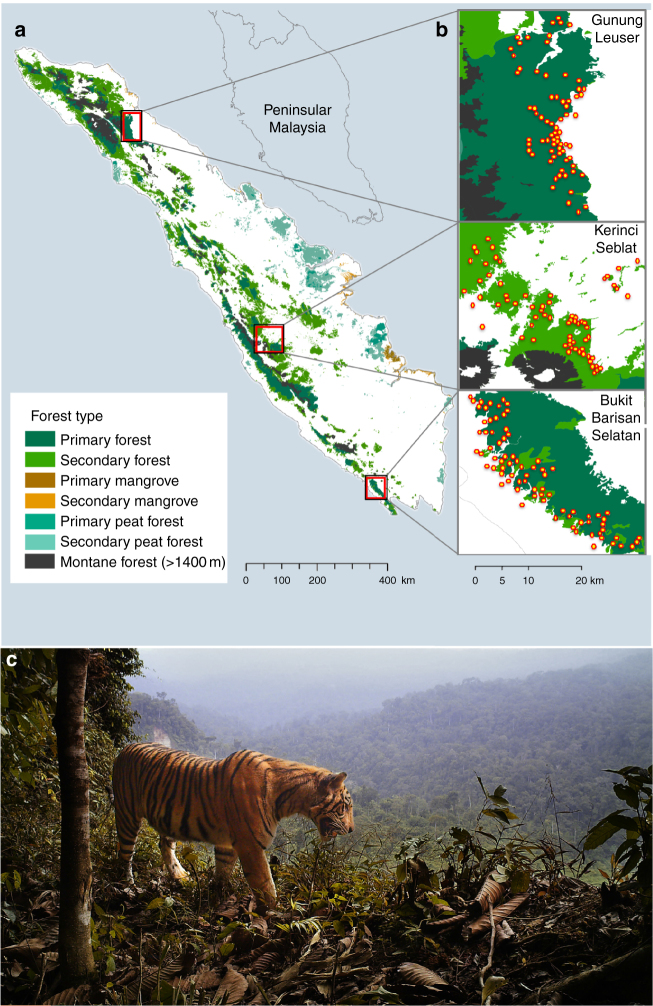
Sumatran tiger habitat and study design. **a** 2012 forest cover in Sumatra, Indonesia. **b** Camera trapping study sites with placement of cameras shown by yellow points. **c** Photo of a Sumatran tiger in Bukit Barisan Selatan National Park showing how a tiger's unique striping pattern is used to identify individuals

Here we develop an approach to characterize spatiotemporal trends in wildlife populations based on CR analyses and apply it to the critically endangered Sumatran tiger (*Panthera tigris sumatrae*). We also assess three questions about Sumatran tiger ecology and conservation: how tiger densities (i) vary between different forest types (i.e., peatland versus lowland forest), (ii) vary in response to habitat disturbance (primary versus degraded forest), and (iii) vary over time. To characterize density variations and estimate populations sizes, we use a five-stage process of linked methods. First, we collated a data set of tiger densities from all published non-spatial CR studies. Second, we recalculate CR densities after adjusting their respective study areas to allow for comparisons to SCR estimates. Third, we add to this set of standardized densities the more recent SCR densities, as well as from three new CT surveys that we conducted in Sumatra’s UNESCO Heritage Site forests in 2014 (Fig. 1, Table 1). Fourth, we compare the results between different forest types and through time using a meta-regression approach that accounts for the error associated with each density estimate. Finally, we extrapolate forest-type specific densities across the island’s remaining tiger-occupied forest area in 2000 and 2012 (Table 2) to estimate subpopulation sizes, total population size, and to identify key sites for conservation.

**Table 1:** Site characteristics and results

	Gunung Leuser	Kerinci Seblat	Bukit Barisan Selatan
*Site characteristics*			
UNESCO protected area (km^2^)	8630	13,753	3568
Contiguous forest 2012 (km^2^)	20,911	18,663	3613
Rainfall (mm)	3000–4650	2500–3500	3000–4000
*Data collection*			
Cameras collected (lost)	69 (13)	73 (23)	78 (5)
Camera days active	3531	5246	5759
Trap elevation (mean ± SD)	313 ± 248	614 ± 197	369 ± 185
Elevation covered (range in m)	27–882	256–1152	114–934
Start of 3-mo survey	December 2013	March 2014	July 2014
Number of fragments (cameras)	3 (4)	8 (24)	4 (4)
CT grid MCP (km^2^)	516	813	474
Effective area sampled (km^2^)	911	999	842
Average camera spacing (m)	1275	1169	1140
*Tiger results*			
Independent captures (individuals)	10 (5)	12 (3)	28 (18)
Tigers ($\hat N$ ± se)	12 ± 7.42	7 ± 6.53	16 ± 7.71
Density in forest ($\hat D$ ± se)	1.32 ± 0.81	0.74 ± 0.65	1.94 ± 0.91

Study sites, sampling effort and area, tiger captures, and human and prey activity in the three national parks that make up the UNESCO Tropical Rainforest Heritage of Sumatra. The effective area sampled here was calculated in SCR models as the core trapping area MCP plus the 2.45 * the *σ* movement parameter. Kerinci Seblat had a highly irregular shape and uneven camera distribution due to sampling in distant fragments

**Table 2:** Forest cover in Sumatran tiger landscapes 2000–2012

		Primary (km^2^)		Degraded (km^2^)			
Tiger landscape	Montane	Lowland and hill	Peat	Lowland and hill	Peat	Totals (×1000)	12-yr deforestation
1—Ulu Masen	4271	599	0	1884	0	6.8	8.8
2—Gunung Leuser	14,332	1890	1	4969	119	21.3	7.6
3—Sibolga & Bt Toru	5521	0	0	2711	23	8.3	4.5
4—Bt Gadis/RimboPanti	4563	579	0	3693	130	9.0	7.8
5—Rimbang Baling & Bt Hari	1659	1710	0	3143	0	6.5	24.5
6—Kerinci Seblat	10,330	3010	0	4017	6	17.4	12.8
7—Bk Barisan Selatan & Bk Balai	3188	1128	0	1397	0	5.7	9.7
8—Bk Tiga Puluh landscaspe	0	0	0	2842	0	2.8	39.1
9—Senepis Buluhala & Giam Siak	0	0	732	87	2309	3.1	40.9
10—Kuala Kampar & Keremutan	0	0	659	108	5500	6.3	38.8
11—Tesso Nilo	0	0	0	740	125	0.9	55.3
12—Bk Dua Belas	0	0	0	391	0	0.4	36.9
13—Berbak	0	0	0	687	2721	3.4	11.7
14—Harapan	0	0	0	1108	0	1.1	7.2
15—Way Kambas	0	0	0	1312	9	1.3	5.1
Remaining forest (×1000 km^2^)	43.9	8.9	1.4	29.1	10.9	94	16.5
Forest loss 2000–2012 (%)	3.9	23.1	35.5	20.5	34.8	16.5	-

Data sources and analyses described in the Methods

The maintenance of ecologically effective densities of apex carnivores is important for the structure and function of natural ecosystems^2^. Globally, tiger populations have declined by over 95% since 1900 and tigers occupy less than 7% of their historical range, split up precariously in small fragmented areas^9–13^. This decline occurred rapidly for Sumatran tigers, the southernmost extant subspecies^12, 14^. Sustained oil palm expansion, forest degradation, and poaching continue to threaten the few remaining tiger populations on the island^12, 15^. Ironically, despite being globally beloved, having a high risk of extinction and being the focus of immense conservation programs, trends in Sumatran tiger densities remain ambiguous^15^. Currently, tiger numbers are usually estimated for specific parks based densities from one or a few studies, each with a large error, and these may not be comparable due to methodological differences^11, 12, 16^. This greatly limits our ability to make island-wide assessments and efficent conservation decisions. This project fills that research gap for the Sumatran tiger while the analytic approach developed here can be widely replicated for other species routinely monitored with capture–recapture techniques.

We find that while tiger densities have significantly increased over the last decade, the disproportionate loss of higher quality lowland and hill primary forest habitat, in combination with severe fragmentation of remaining strongholds, has offset this important conservation achievement and led to an equivocal or higher threat of extinction. We derive habitat-specific tiger densities and use past and current forest cover to estimate the change in tigers in each of Sumatra’s remaining occupied landscapes. We conclude by discussing the imminent and often irreversible threat of deforestation and fragmentation compared to the previous dominant threat of poaching, with a specific focus on the role of oil palm agriculture in driving forest loss.

## Results

### Standardizing CR density estimates

**Fig. 2:**
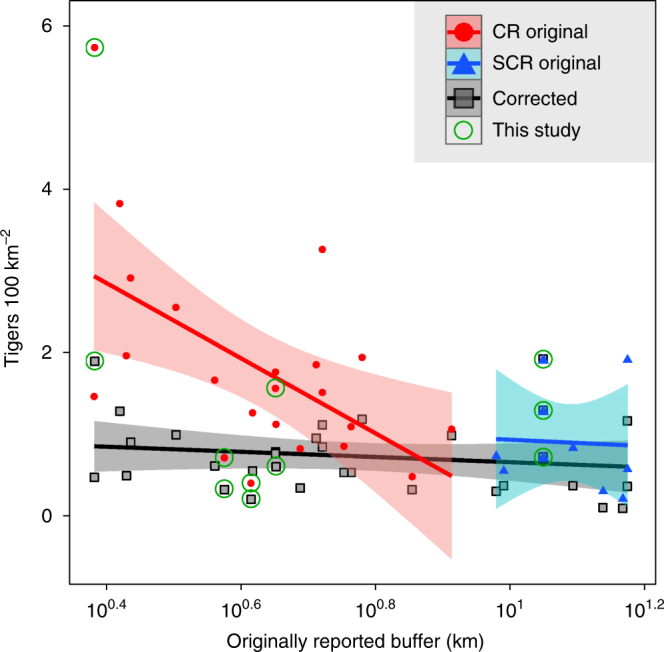
Tiger density bias due to the use of camera trap grid buffers. Tiger densities reported for Indonesian and Malaysian rainforests as a function of the originally reported buffer width used to calculate the effective area sampled (*n* = 29 estimates from 13 forests). Data point shapes denoted by the analysis method used to estimate density (circles for CR, triangles for SCR, squares for corrected densities, the latter recalculated using a standard buffer length of 9.0 km derived from the new SCR analyses in this study). Red indicates CR studies, blue for SCR studies, and gray for corrected densities

To characterize temporal and cross-site trends in tiger densities in tropical rainforests, we collated a set of previously published tiger studies and added our three new estimates from 2014. Recalculating CR densities proved crucial to interpreting results from across Sumatra (*n* = 20 estimates from 11 landscapes) and also nearby in ecologically similar tropical rainforests in Peninsular Malaysia (*n* = 6 estimates from four landscapes). Using uncorrected density estimates incorrectly suggested that tiger densities declined 67.0% in publications post 2010. This decline is in fact due to the switch from CR to SCR methods; densities actually increased post 2010 after we recalculated CR estimates to remove the study area bias^5^ (Supplementary Fig. 1). The CR bias arises from difficulties in sufficiently accounting for animals captured in CT grids that have home range centers in the buffer areas around grids (see Methods for details). For a given abundance of animals overlapping with CT grids (*N*), a smaller CR buffer leads to smaller study areas and higher densities (Supplementary Fig. 2). Buffer widths were significantly smaller for CR studies than for SCR studies (mean 6.30 versus 11.7 km, Welsh two-sample *t*-test (WT), df = 14, *P* < 0.01). Raw tiger densities were most strongly dependent on estimated buffer widths (Linear regression: *R*^2^ = 0.43, *P* < 0.01), and not on any underlying ecology (Fig. 2). To remove this bias, we recalculated all CR densities by digitizing camera trap grids in ArcGIS and then applying the SCR buffer from our fieldwork (buffer = 9.0 km, see Methods). CR densities declined from an uncorrected mean of 2.16 tigers/100 km^2^ to a corrected mean of 0.79 tigers/100 km^2^ (WT_14_: *P* < 0.01) and the approach was deemed effective as there were no differences between CR and SCR estimates after the correction (Fig. 2).

### New SCR density estimates

Our new fieldwork was undertaken as part of an effort to assess the contested degree to which tigers can coexist with humans in both space and time (e.g., Carter et al.^17^ and responses). Specifically, we surveyed both continuous protected forests (national parks) and adjacent non-protected areas, mostly consisting of a matrix of tree plantations and small (<5 km^2^) forest patches (Fig. 1, Table 1). Our trapping effort was 5759, 5246, and 3531 camera days in Bukit Barisan Selatan (BBS), Kerinci Seblat (KS), and Gunung Leuser (GL) national parks, respectively (Fig. 1, Table 1), which is sufficient to estimate tiger densities (Supplementary Fig. 3). We report the SCR tiger densities as mean ± 1 standard error. Densities in BBS, KS, and GL were 1.94 ± 0.91 tigers/100 km^2^, 0.74 ± 0.65 tigers/100 km^2^, and 1.32 ± 0.81 tigers/100 km^2^, respectively. Densities in non-forest habitats were 0.56 ± 0.93, 0.21 ± 0.36, and 0.38 ± 0.78 tigers/100 km^2^ in GL, KS, and BBS, respectively (71% lower than continuous forest on average and also presumably contingent on there being continuous forest nearby). This indicates that while tigers regularly use human-dominated landscapes immediately outside national parks, this is a relatively poor habitat.

### Habitat and temporal trends

**Fig. 3:**
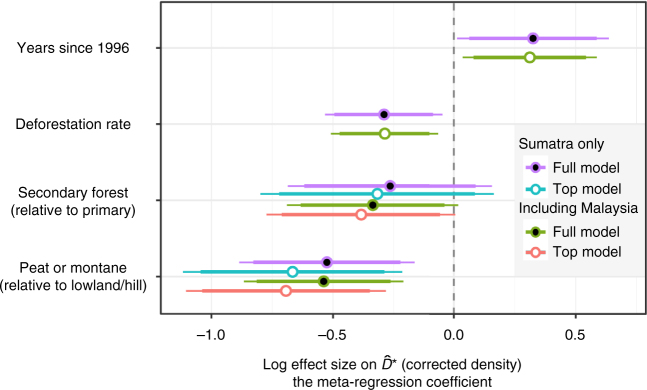
Variation in tiger densities through time and between habitats. Results from mixed model meta-regressions explaining variation in log($\hat D$*) showing 90% CI (thick lines) and 95% CI (thin lines). Top model (white fill) based on AICc values included habitat and degradation (secondary forest). Full model (black fill) including time and deforestation rates had marginally higher AICc values (+3.3). Malaysia estimates were not significantly different than Sumatra’s accounting for forest type and disturbance (LRT: *χ*_1_ = 0.67, *P* = 0.41) and the top Sumatra-only model also included forest type and disturbance (LRT_S_: *χ*_2_ = 7.93, *P* = 0.02, *R*^2^ = 0.50) and supported lower densities in peat and montane forests (−48.6%; MRMM_S_: *z* = −2.90, *P* = 0.01), and logged areas (−27.2%; MMRM_S_: *z* = 1.29, *P* = 0.21). Including deforestation and time significantly improved the Sumatra-only model fit (LRT_S_: *χ*_2_ = 8.65, *P* = 0.01, *R*^*2*^ = 0.77) and indicated densities increased from 2000 to 2012 by 47.6% (MMRM_S_: *z* = 2.04, *P* = 0.07). All models included a random effect to account for multiple estimates from the same landscape

We constructed meta-regression mixed models (MRMM) to evaluate if tiger densities meaningfully varied with site or study covariates, while accounting for differences in the precision of density estimates and including a random effect to account for multiple estimates nested in the same landscape (*n*_S_ = 20 estimates from 11 landscapes). We include estimates from Malaysia since the habitat conditions are similar to Sumatra (*n*_M_ = 6 estimates from four landscapes; model subscripts denote ‘S’ for Sumatra and ‘S&M’ when including Malaysian estimates). Including Malaysian estimates only reinforced results from Sumatra, thus we reported results in the main text using all available data and include Sumatra-only results in Fig. 3. Due to the massive areas covered by many CT grids, we conservatively separated study landscapes into two forest type and two forest disturbance categories, and looked at temporal trends for both logged and unlogged forests (Supplementary Fig. 4). We grouped lowland and hill forests (thought to be prime tiger habitats) and compared these to peat and montane (>1000 m) forests and we compared predominately primary forest landscapes to areas with substantial logging (Fig. 3). Including forest type and disturbance provided the most parsimonious informative model of variation in tiger density (likelihood ratio test (LRT)_S&M_: *χ*_2_ = 12.92, *P* < 0.01; *R*^*2*^ = 0.52; AICc declined by 1.86 compared to models with fewer or more variables). Densities in peat and montane forests were significantly lower than in lowland and hill forests (−49.9%; MRMM_S&M_: *z* = −3.31, *P* = 0.01), as suggested in other work^16, 18^. Densities were 31.9% lower in landscapes with substantial logging compared to continuous primary forests (MRMM_S&M_: *z* = 1.93, *P* = 0.07). Including time (years since first CR study in 1996) and deforestation rates significantly improved model fit (LRT_S&M_: *χ*_2_ = 9.63, *P* = 0.01, *R*^*2*^ = 0.77), but led to slight overfitting (AICc increased by 3.3). As a result, we report these full model results directly, but do not include these covariates when estimating tiger population sizes. The full model indicated that tiger densities significantly increased from 2000 to 2012 by 46.4% total (4.9%/yr; MMRM_S&M_: *z* = 2.22, *P* = 0.04; Sumatra only: *P* = 0.07). Densities were 14.5% lower in landscapes for every 1% increase in annual deforestation (MMRM_S&M_: *z* = 2.5, *P* = 0.03).

### Tiger subpopulation sizes

**Fig. 4:**
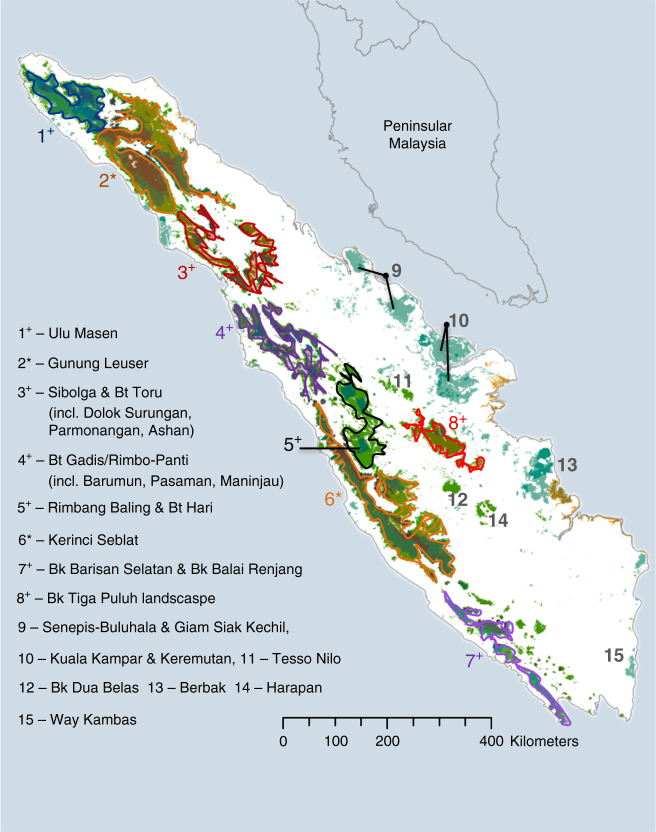
Remaining tiger landscapes and source populations. Landscapes with >25 breeding females are indicated with asterisk (*) and other important landscapes are indicated with the “+”. Bt = “Batang”, and Bk = “Bukit”. Colors only used to aid visual differentiation of landscapes

We estimated populations in tiger-occupied forests^19^ using existing habitat maps^20^ for 2000 and 2012 and the habitat-specific tiger densities derived from the top meta-regression model (which did not include time or deforestation rates). The global tiger conservation strategy focuses on maintaining a network of secure source populations (SSP) that have >25 breeding females and “are embedded in a larger landscape with the potential to contain >50 breeding females”^11^. We report tiger subpopulation estimates as mean ± standard error. The only SSPs remaining were Gunung Leuser (140 ± 66 tigers) and Kerinci Seblat (122 ± 57 tigers; Table 3). Three other potential SSPs had a mean estimate of 18–21 breeding females: the Sibolga and Batang Toru landscape, the Batang Gadis and Rimbo-Panti landscape, and the Rimbang Baling and Batang Hari landscape, with a combined total of 165 ± 77 tigers. Ulu Masen (45 ± 21 tigers) remains important because it is connected to Gunung Leuser. The greater BBS landscape (41 ± 19 tigers), including the Tambling Wildlife Nature Conservation to the south and Bukit Balai to the north, remains important because of high densities and strong anti-poaching efforts (Fig. 4). The estimated total abundance in these seven priority landscapes is 514 ± 241 tigers. The island-wide population estimate, including smaller forests and rapidly vanishing forests, is 618 ± 290 tigers (Supplementary Table 1). Using the same method and assuming that densities did not vary with time, we estimate that there were 742 ± 348 tigers in 2000 and that land use change reduced the potential Sumatra tiger population by 16.7% in just 12 years. By comparison, multiplying the full 2012 tiger-occupied forest area by the uncorrected mean of all Sumatran tiger density estimates (including CR and SCR studies) produced naive island-wide estimates of 1804 and 1507 tigers in all forests in 2000 and 2010, respectively (Supplementary Table 2).

**Table 3:** Sumatran tiger population estimates in 2000 and 2012

Tiger forest landscape	Adult tigers 2012	Adult tigers 2000	Breeding females 2012	Breeding females 2000	Tigers −1se	Tigers +1se
1—Ulu Masen	45	51	16	17	24	66
**2**—**Gunung Leuser**	**140**	**154**	**48**	**53**	74	206
3—Sibolga & Bt Toru	52	54	18	18	27	76
4—Bt Gadis/Rimbo-Panti	61	67	21	23	32	89
5—Rimbang Baling & Bt Hari	53	71	18	25	28	77
**6**—**Kerinci Seblat**	**122**	**145**	**42**	**50**	65	180
7—Bk Barisan Selatan	41	46	14	16	22	61
8—Bk Tiga Puluh	22	36	7	12	12	32
9—Senepis-Buluhala	14	23	5	8	7	20
10—Kuala Kampar	26	42	9	15	14	38
11—Tesso Nilo	6	13	2	5	3	9
12—Bk Dua Belas	3	5	1	2	2	4
13—Berbak	16	17	5	6	8	23
14—Harapan	8	9	3	3	4	12
15—Way Kambas	10	10	3	3	5	15
Totals	618	742	91	103	328	908

Estimated by multiplying the habitat-specific densities by the area remaining of each habitat type in each tiger landscape. Source populations are defined as those with 25 breading females and are shown bolded. ‘Bt’ denotes Batang and ‘Bk’ denotes Bukit. For delineating landscapes here, Rimbang Baling & Bt Hari includes Bk Bungkok, Bk Barisan Selatan includes Bk Balai, Bk Tiga Puluh includes Peranap, Senepis-Buluhala includes Giam Siak, and Kuala Kampar includes Keremutan

## Discussion

The decline in Sumatran tiger SSPs was driven by rapid loss and selective logging of continuous large habitats. Our analysis suggests that of the 12 SSP potentially existing in the mid-20th century^11, 15^, only two now exceed >25 breeding females as of 2012. The decline of SSPs was not due to changes in tiger densites from poaching or prey depletion, as mean tiger densities actually increased from 1996 to 2014 (Fig. 2). Instead, between 1990 and 2010, 37% of Sumatra’s total primary forest was lost^14^, and from 2000 to 2012, tiger-occupied forests declined 16.5%. Further, forest loss disproportionately affected high tiger density landscapes such as primary lowland and hill forest (Table 2). Lowland and hill forest area declined 21.1%, largely due to the expansion of palm oil plantations that drove annual deforestation rates >3% in Jambi, Riau, South Sumatra, and Benkulu provinces (i.e., 30–60% total forest cover declines in Bukit Tiga Puluh, Senepis-Buluhala and Giam Siak, Tesso Nilo, and Bukit Dua Belas landscapes; Table 2). Meanwhile, montane forests with lower tiger densities only declined 3.4%. Tiger densities were 31.9% lower in disturbed (logged) areas and 80.0% of Sumatra’s remaining hill, lowland, and peat forest is already disturbed (as of 2012). Taken together, swift and effective conservation efforts to control deforestation and forest degradation is necessary, lest Sumatran tigers meet the same doomed fate as the Balinese and Javanese tiger sub-species that went extinct during the 20th century^9^.

The Gunung Leuser and Kerinci Seblat SSPs are crucial to the long-term persistence of Sumatran tigers in the wild. Provided future deforestation and poaching is controlled, each of these parks could maintain >40 breeding females and likely persist without supplemental breeding programs for >200 years^13^. Unfortunately, that rosy situation is unlikely in Kerinci Seblat were 12.8% of the forested area was lost from 2000 to 2012 and poaching continues despite intensive patrolling ^21^. Gunung Leuser’s population is the most robust with lower deforestation rates and good connectivity to Ulu Masen. Short-term conservation efforts to save other potential SSPs should focus on the Rimbang Baling/Batang Hari landscape that is connected to Kerinci Seblat, but where deforestation exceeded 20% from 2000 to 2012, while longer-term conservation should also include the Sibolga/Bukit Toru and Bukit Barisan Selatan landscapes.

We acknowledge that  there are always trade-offs between different approaches to estimating population sizes. Our island-wide range of 328–908 tigers is slightly higher than the range of 441–679 reported by the IUCN assessment^15^. Although we used many of the same raw density estimates as the IUCN, we corrected inflated CR estimates and, instead of using site-specific densities to separately estimate each forest’s tiger population, we used island-wide habitat–density relationships based on a meta-regression that provides a standardized measure of error. We suggest our approach is better suited to population projections when large portions of the species’ occupied habitat is unsurveyed and when wide confidence intervals make reliance on any one density estimate less suitable, both of which are true for tigers in Sumatra^11, 15^, but less so in regions with greater research efforts (e.g., tigers in India, but see ref. [9]). Our habitat- and degradation-specific densities are also suitable to re-estimating populations as new land cover data becomes available and for scenario building. Finally, we highlight that the meta-regressions can easily be updated with new density estimates to facilitate a methodologically consistent island-wide monitoring program through time, and time itself can be a covariate.

We urge cautious interpretation of the result that tiger densities increased from 1996 to 2014 because the data set comprises different sites through time. Further, it is possible the analysis is capturing a recovery from all-time low densities following extensive poaching in the 1980s and 1990s^10, 20^ and densities are unlikely to continue increasing. That explanation is supported by our data set including many comparatively well-protected sites. Camera trapping itself is often part of tiger monitoring and anti-poaching programs, so there is a definite bias in the dataset^21^. Finally, a rise in density may be caused by the inward movement of encroachment-displaced tigers from forest edges or fragments. Regardless of whether the apparent increase in tiger densities in core areas represents a real recovery or is due to a sampling artefact, any potential increase therein has been largely offset by the habitat loss and unlikely to have led to larger island-wide populations over the study period.

Our final insight relates to how populations are monitored through time. The inability to draw inferences across CR studies has been an important limitation in predator ecology and conservation. Optimally, precisely repeated surveys using the same field and analytical methodologies would be conducted often, but this is rare in practice. Our approach to standardize between non-spatial and spatial CR can statistically control for basic differences, but reanalyzing the raw capture data in an multi-site SCR framework would be preferable^22^. Our cross-site meta-regression approach to evaluating variation in animal densities between habitats and through time yielded a more geographically expansive and longer-term perspective on our study species than previously available. Wider application of this approach can substantially increase the value of previous CR studies, but would still benefit from more total density estimates. We foresee the approach outlined here being repeated for other elusive species and leading to methodologically consistent and defensible monitoring programs.

## Methods

### Locations of density estimates

Our Sumatra-wide tiger density data set comes from two sources: first, we compiled all published density estimates for Sumatra and details of their study design, location, and analysis approach (*n* = 17 estimates from eight landscapes; Supplementary Table 3). We also included six density estimates from four tropical lowland or hill rainforests in Peninsular Malaysia. Second, we generated three SCR estimates from our own fieldwork (Table 1).

### Study sites

New tiger densities were estimated from CT surveys in 2014 at Gunung Leuser National Park (GL), Kerinci Seblat National Park (KS), and Bukit Barisan Selatan National Park (BBS) (Fig. 1). These national parks together constitute UNESCO’s “Tropical Rainforest Heritage of Sumatra,” and are nationally and internationally protected^23^. The vegetation is wet evergreen tropical rainforests, and we sampled both lowland and hill forests with rainfall from 2500 to 4700 mm and temperatures between 22 and 35 °C (Table 1). All sites are bordered by a mix of industrial and smallholder plantations of oil palm, rubber, rice, coffee, and cacao, in that order of decreasing abundance. To estimate densities in the adjacent bordering mixed-use habitats, we sampled 11 forested ridgelines in fragments remaining in the converted landscapes.

### Data-collection procedures for new density estimates

We deployed 69–78 passive infrared camera traps for a 2–3-month period set across areas of 474–813 km^2^ grids at each park (Fig. 1B; Table 1). The cameras were placed within pre-mapped 1.5 km × 1.5 km grids and spaced >1 km apart. To standardize deployment between sites, all cameras were placed along ridgeline wildlife trails at elevations 50–1200 m above sea level (asl). To estimate densities within continuous protected forests, we sampled at distances of 0–15 km into forest interiors. We used a single camera placement at each trapping location, versus paired cameras, which reduced the probability that all captured animals can be identified using both flanks, but partially made up for this shortcoming by collecting multiple photos per capture event (i.e., sequences or videos) to increase recognition of non-flank features (i.e., facial, chest, legs, or tail markings). We obtained 50 independent captures of tigers across all three sites and identified 26 unique individuals.

### Capture–recapture analyses

To estimate density from CT data, tigers are identified by their unique coat markings and capture histories are created for each individual^24^. In the conventional non-spatial CR approach, the abundance ($\hat N$) of animals in the trapping area is estimated using a closed-population CR model^24, 25^. Density ($\hat D$) is then derived by dividing $\hat N$ by the estimated effective area sampled, ($\hat A$), defined as the minimum convex polygon (MCP) around the CT perimeter plus a buffer. Buffers are used to account for captures of animals with home range centers outside the CT grid and the widths of buffers are derived from the distances between recaptures of the same animals at different cameras. For example, the most common type of CR buffer used is half the mean maximum distance moved (1/2 MMDM). However, small CT grids maybe unlikely to fully capture the largest animal movements and it also is unlikely for recaptures to occur at opposite edges of an animal’s home range^5^. As a result, the traditional CR approach systematically underestimates animal movements, and thus buffer widths and the effective area sampled $\hat A$^5^. When $\hat A$ is biased down (underestimated), $\hat D$ is biased high.

### Spatial capture–recapture

Spatial capture–recapture (SCR) builds on traditional CR approaches^26, 27^ and has gained favorability because SCR eliminates the use of subjective buffers^5^. The location of individual home range centers is modeled based on the spatial information of captures such that $\hat D$ itself is implicitly derived from the analysis. SCR also includes an animal movement parameter (*σ*), which is the standard deviation of the half-normal distribution used to specify change in detectibility (*g*0) with distance from home range centers. SCR can be implemented in both likelihood and Bayesian frameworks and $\hat D$, *g*0, and *σ* can be modeled as a function of covariates such as season, site, habitat, or sex^28^.

### SCR analyses for new density estimates

We used SCR models to estimate the density of tigers in GL, KS, and BBS^26, 28^. We included habitat masks^29^ to exclude water bodies (i.e., the Pacific Ocean) and separately model densities inside and outside continuous forests. To overcome the limitation of low numbers of individuals at each site, we analyzed all three sites together in multi-session model framework^28^, where a single model shares information from all sites to help estimate parameters (i.e., *g*0 and *σ*). For GL and KS forests, we were able to identify and analyze both right and flank photos together because enough tigers turned in front of CTs to identify their coat patterns on both sides. For BBS, however, we were unable to create a single-capture history. Within our multi-session model, we included the BBS left and the right-flank captures as different trapping sessions while constraining $\hat D$, *g*0, and *σ* parameters to be the same for both sessions. We implemented the SCR models in a maximum-likelihood framework using the secr package (version 2.10.3) in R (version 3.2.0)^29^. The final model selection did not support including sex- or site-specific parameters. We calculated home range size as the 95th percentile of a half-normal distribution scaled by sigma (*σ* = 4.59 ± 0.95 km). This produced a tiger home range size of 397 km^2^, which is similar to that found in other SCR studies (mean *σ* = 5.33) and within the bounds of observed in telemetry work^30^.

### Approach to standardizing density estimates

As described earlier, CR buffers were systematically underestimated^5^, so we recalculated them using a standard larger buffer derived from our SCR analysis. The corrected densities are still derived using the CR approach for calculating $\hat N$, the key point is that $\hat D$*_CR_ is improved because we borrow information from SCR results to estimate the buffer width, thus improving $\hat A$ compared to MMDM approaches to estimating buffers and $\hat A$. Using the three sites we surveyed, we estimated the new buffer as the 95th percentile of the half-normal distribution used to describe the home range in the SCR model (σ*1.96, equal to 9.0 ± 1.86 km). There was insufficient information on variation in tiger movements in Sumatra to include sex-specific, habitat-specific, or site-specific *σ* or buffers. We manually digitized CT locations from published studies in ArcGIS and calculated the corrected effective area sampled using the new buffer ($\hat A$*; Supplementary Tables 3–5). We calculated the corrected density $\hat D$*_CR_ by dividing the originally reported adult tiger abundance $\hat N$ by $\hat A$*, and derived the standard error for $\hat D$*_CR_ from that of $\hat N$, as is normal in all CR studies^24^. As the original CR buffers were consistently smaller than 9.0 km, $\hat D$*_CR_ was lowered. The effectiveness of this bias-correction method is supported by simulation work^5^ and Supplementary Fig. 5 and can be explored using R code provided in Supplementary Data 1 of this paper. Nonetheless, corrected estimates sometimes deviate from SCR results for sparse data sets because the SCR analyses pooled data to estimate shared parameters (*σ*, *g*0) and produced habitat-specific densities (all our results are presented in Supplementary Table 3).

### Covariates of tiger density

We used meta-regression mixed-models (MRMMs) to evaluate if variation in standardized tiger densities ($\hat D$*_CR_ and $\hat D$_SCR_, hereafter $\hat D$*) was explained by habitat type, disturbance level, or time trends. The sampling distribution of unique individuals (*M*_+1_) identified in a given study area is asymptotic causing $\hat N$ and $\hat D$ to follow a lognormal distributions^31^. Further, for given study areas, more data (higher *N* and more recaptures) improves model accuracy and precision. As a result, the relative standard error (s.e. as a proportion of the actual estimate) decreases as densities increase, and the s.e.(log($\hat D$*)) is derived as s.e.($\hat D$*)/$\hat D$*. We set up our MRMMs with log($\hat D$*) as the response variable and weighted estimates based on their relative s.e. using the lme4 package in R^32^. To reduce effects of spatial and temporal pseudo-replication, we accounted for multiple estimates from the same tiger landscape by including a random effect for “landscape” in all analyses. The following variables were tested as predictors of the standardized tiger density in the MRMMs: year sampling occurred, forest size (as reported by original study), forest type (lowland and hill >1000 m asl), montane (predominantly >1000 m asl), disturbance (predominantly primary versus areas with logging), deforestation rate (as reported in ref. ^12^), sampling effort (number of operational trap days), number of cameras deployed, tiger captures, and the number of tiger individuals identified (Supplementary Data 1). Model selection was done with ANOVA Chi-squared test and AICc, and model fit was evaluated by marginal *R*^2^ for mixed models^33, 34^ (Table 2).

### Secure source populations and total population size

We defined SSP as landscapes with a large core forested areas (>1000 km^2^) holding >25 breeding females (bf), and which is nearby to other forests containing another 25 bf^11^. This is supported by recent simulations that show populations under 25 bf face high extinction rates over long time horizons due to genetic and stochastic effects, and are particularly vulnerable to poaching^13^. We identified tiger-occupied landscapes following Wibisono et al.^19^, and estimated forest cover in each landscape in 2000 and 2012 using high-resolution land cover data^20^, and report 12-yr deforestation rates as the difference between time periods (our values closely matched those of another land use change analysis for Sumatran tiger landscapes, i.e., ref. ^12^; Fig. 1; Table 3). We estimated abundances in each landscape by multiplying the habitat-specific densities by the extent of forest in each category. We estimated the number of bf using the ratio established for camera trap studies (1 bf in 2.9 total tigers in ref.^13^). We report the s.e.’s based on the mean proportion of se to mean estimate from the data set of standardized densities.

### Data availability

As with many endangered species, to prevent sensitive locality information being used by poachers, the convention is to limit publication of raw capture data. However, all data and code is available from the authors upon email request.

## Electronic supplementary material

Supplementary Information

Description of Additional Supplementary Files

Supplementary Data 1
