# Publisher Correction: Sumatran tiger survival threatened by deforestation despite increasing densities in parks

**DOI:** 10.1038/s41467-018-03055-9

**Published:** 2018-02-12

**Authors:** Matthew Scott Luskin, Wido Rizki Albert, Mathias W. Tobler

**Affiliations:** 1 Center for Tropical Forest Science – Forest Global Earth Observatory, Smithsonian Tropical Research Institute, PO Box 37012, Washington, DC 20012 USA; 2 Asian School of the Environment, Nanyang Technical University, 50 Nanyang Avenue, N2-01c-36, Singapore, 639798 Singapore; 3 Department of Environmental Science, Policy, and Management, University of California, Berkeley, 204 Mulford Hall, Berkeley, CA 94720 USA; 4 Fauna & Flora International - Indonesia Programme, PO Box 42, Sungai Penuh, Kerinci, Jambi,, 37111 Sumatra Indonesia; 5 San Diego Zoo Global, Institute for Conservation Research, 15600 San Pasqual Valley Road, Escondido, CA 92027-7000 USA

Correction to: *Nature Communications* 10.1038/s41467-017-01656-4, published online 05 December 2017

In the original version of the Article, reference 18 was incorrectly numbered as reference 30, and references 19 to 30 were incorrectly numbered as 18 to 29. These errors have now been corrected in the PDF and HTML versions of the manuscript.

