# Supplementary material for: Sumatran tiger survival threatened by deforestation despite increasing densities in parks
Source: Nat Commun. 2017 Dec 5;8:1783. doi: 10.1038/s41467-017-01656-4 (PMC5717059; doi:10.1038/s41467-017-01656-4)
Supplement: Supplementary file 1 — Supplementary Information [file 41467_2017_1656_MOESM1_ESM.pdf]

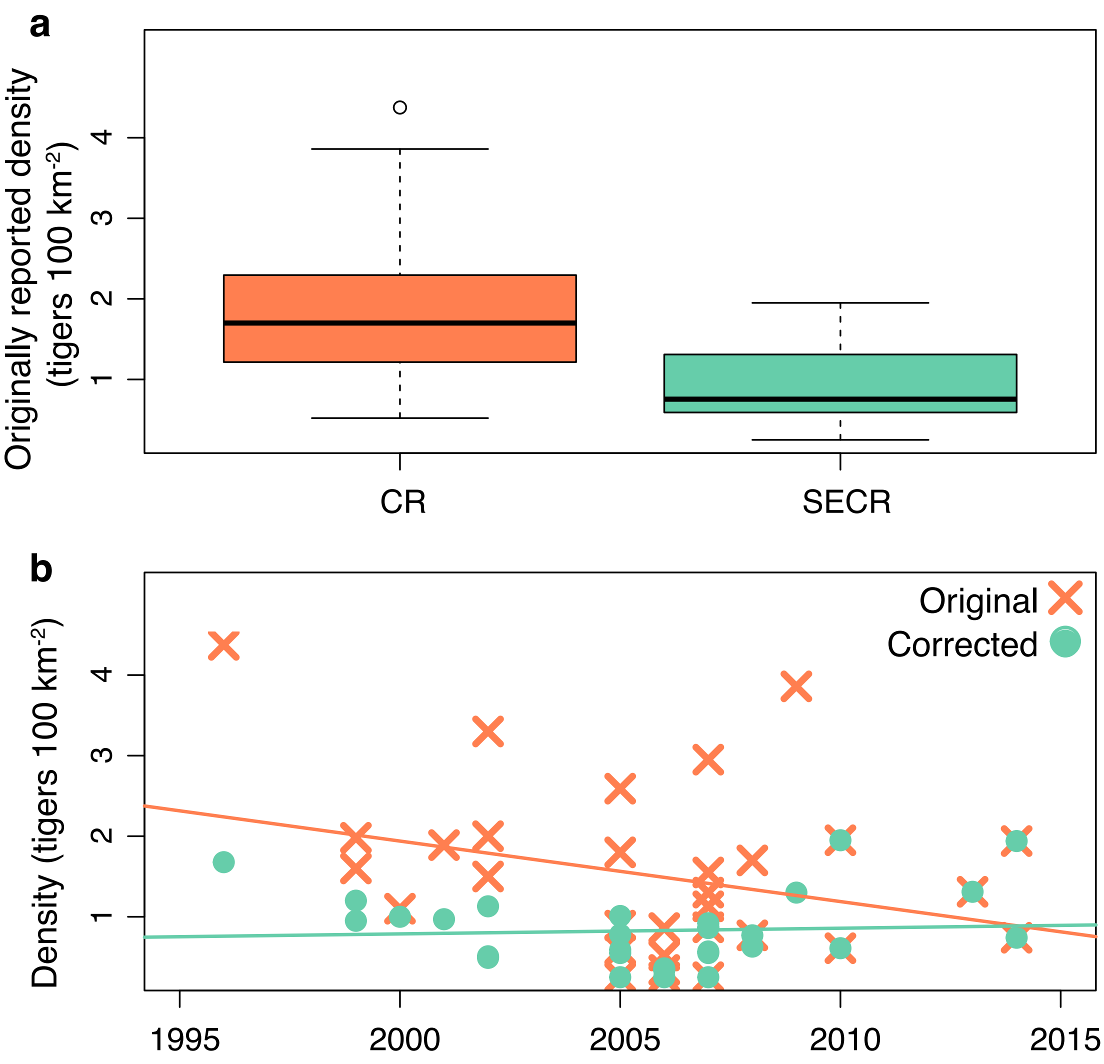


**Supplementary Figure 1 | Relationships between original and corrected densities.** (a) Originally reported Sumatran tiger densities. (b) Originally reported and corrected Sumatran tiger densities through time (SCR estimates were not corrected and contribute to both regression lines).

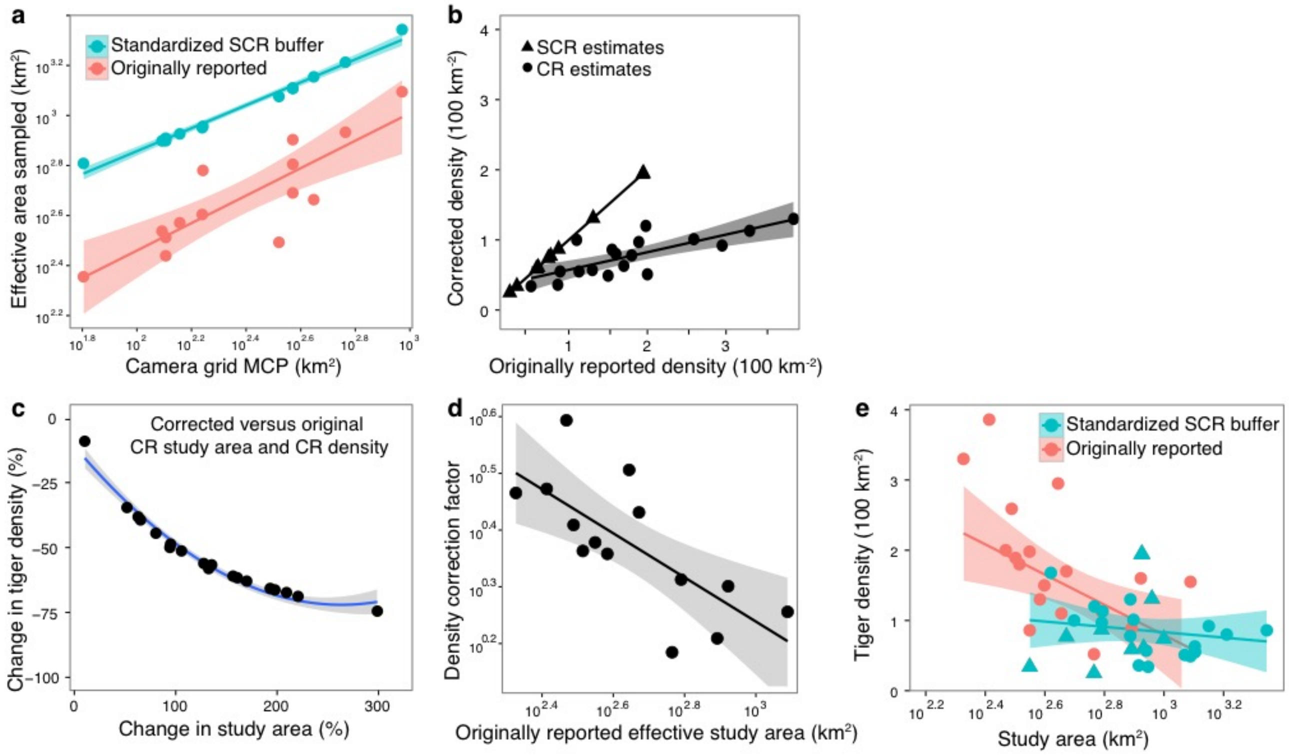


**Supplementary Figure 2 | Relationships between original and corrected study areas and densities.** 'Density correction factor' is the corrected density divided by the originally reported density.

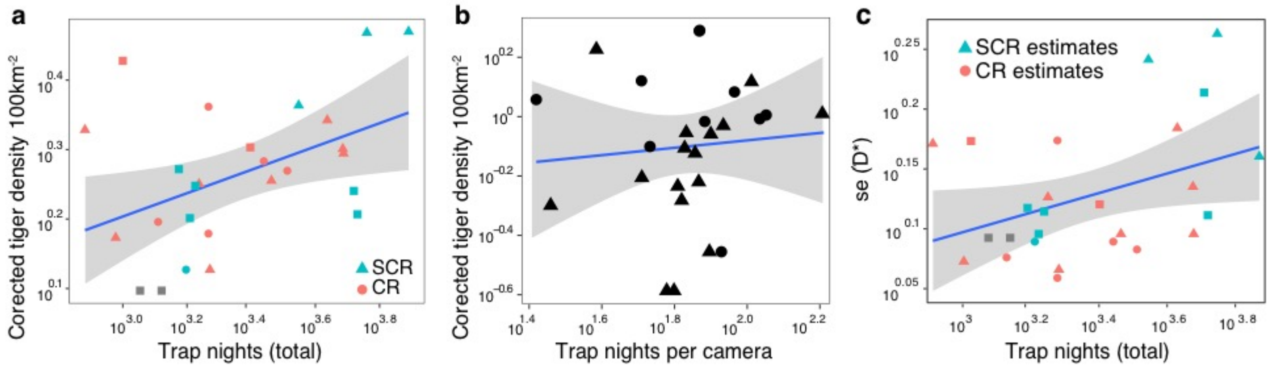


**Supplementary Figure 3 | Relationships between sampling effort and corrected densities.** Sampling effort is measured as total trap nights (sum over all cameras) or mean trap nights per camera. Increasing trapping effort, more often through the use of more cameras, is associated with higher tiger densities (a) and larger error (c), although these two variables are also positively correlated. There is no relationship between trap nights per camera and densities (b).

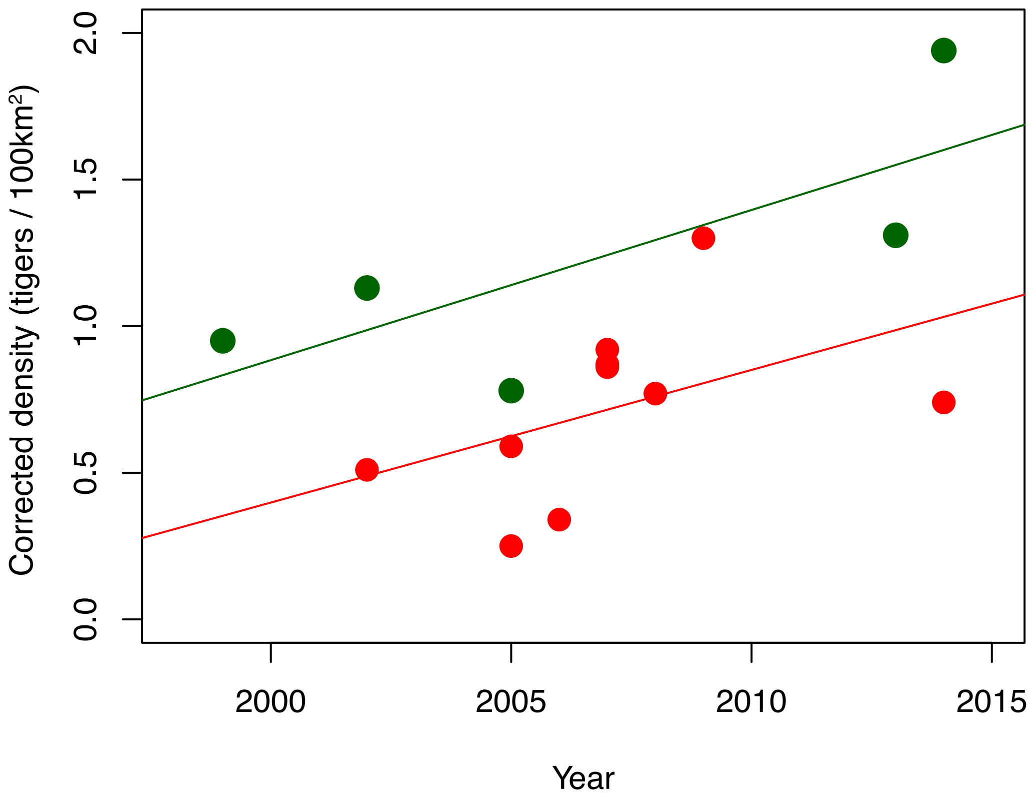


**Supplementary Figure 4 | Corrected tiger densities through time in logged and unlogged Sumatran lowland forests.** Green points and trend line shows primary sites, red points and trend line show sites where there is logging.

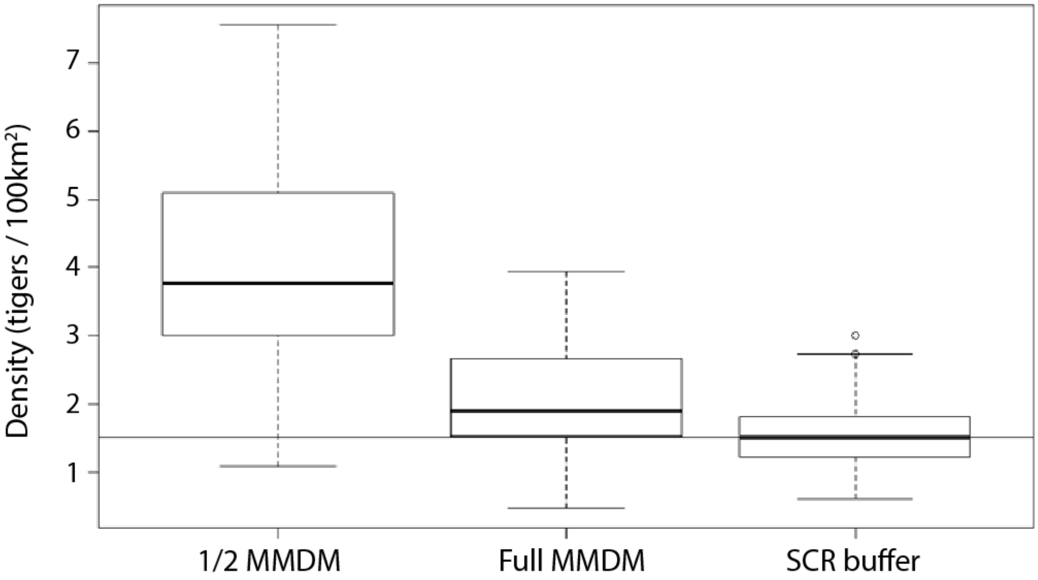


**Supplementary Figure 5 | Densities derived for the same 50 simulated datasets.** The solid horizontal line is the 'true' density we set in the simulation (1.5 tigers/100km$^2$). Boxploxs of results are shown for the traditional Mh model (closed-population jackknife estimator). CR densities vary based on the specific approach used to estimate buffer width when determining the effective trapping area $\hat{A}$(W), which is the MCP plus a buffer. Here we show results for two prominent CR buffers approaches: ½ MMDM and Full MMDM, as well as our approach of using a buffer derived from the 95% home ranges calculated using the SCR sigma parameter. The code for reproducing simulations and results is provided in Supplementary Table 5. Applying the SCR buffer when re-calculating CR densities produced unbiased results.

| Tiger landscape | This study | Joshi *et al* |
|---|---|---|
| **1** – Ulu Masen | 8.8 | 6.8 |
| **2** – Gunung Leuser | 7.6 | 6.8 |
| **3** – Sibolga & Bt Toru | 4.5 | 3.1 |
| **4** – Bt Gadis/RimboPanti | 7.8 | 3.2 |
| **5** – Rimbang Baling & Bt Hari | 24.5 | 26.8 |
| **6** – Kerinci Seblat | 12.8 | 14.3 |
| **7** – Bk Barisan Selatan & Bk Balai | 9.7 | 9.4 |
| **8** – Bk Tiga Puluh landscaspe | 39.1 | 50.3 |
| **9** – Senepis Buluhala & Giam Siak | 40.9 | 33.7 |
| **10** – Kuala Kampar & Keremutan | 38.8 | 33.7 |
| **11** – Tesso Nilo | 55.3 | 46.4 |
| **12 –** Bk Dua Belas | 36.9 | 50.3 |
| **13** – Berbak | 11.7 | 8.5 |
| **14** – Harapan | 7.2 | 8.5 |
| **15** – Way Kambas | 5.1 | 3.2 |

**Supplementary Table 1 | Comparison of deforestation rates.** Joshi et al. (2016) rates based on rates from 2001-2014[1]. The insignificant differences between studies could arise from how tiger landscapes demarcated, methods for defining forest cover, and from deforestation occurring in years 2000, 2013, and 2014 that was included in the Joshi et al. (2016) study period.

| Landscape | Forest 2000 | Forest 2010 | Tigers 2000 | Tigers 2010 |
|---|---|---|---|---|
| 1 – Ulu Masen | 7.408 | 6.754 | 119 | 108 |
| 2 – Gunung Leuser | 23.061 | 21.311 | 369 | 341 |
| 3 – Sibolga & Bt Toru | 8.645 | 8.255 | 138 | 132 |
| 4 – Bt Gadis/Rimbo-Panti | 9.724 | 8.965 | 156 | 143 |
| 5 – Rimbang Baling & Bt Hari | 8.629 | 6.512 | 138 | 104 |
| 6 – Kerinci Seblat | 19.92 | 17.363 | 319 | 278 |
| 7 – Bk Barisan Selatan & Bk Balai | 6.326 | 5.713 | 101 | 91 |
| 8 – Bk Tiga Puluh landscaspe | 4.666 | 2.842 | 75 | 45 |
| 9 – Senepis-Buluhala & Giam Siak | 5.289 | 3.128 | 85 | 50 |
| 10 – Kuala Kampar & Keremutan | 10.232 | 6.267 | 164 | 100 |
| 11 – Tesso Nilo | 1.936 | 0.865 | 31 | 14 |
| 12 – Bk Dua Belas | 0.62 | 0.391 | 10 | 6 |
| 13 – Berbak | 3.86 | 3.408 | 62 | 55 |
| 14 – Harapan | 1.194 | 1.108 | 19 | 18 |
| 15 – Way Kambas | 1.257 | 1.321 | 20 | 21 |
| Totals | 112.8 | 94.2 | 1804 | 1507 |

**Supplementary Table 2 | Uncorrected Sumatran tiger population estimates in 2000 and 2010.** Densities are on the mean uncorrected density estimates originally reported from Sumatra for all CR and SCR estimates to date (1.876 tigers $100km^{-2}$). Forest cover in 2000 and tiger occupied forests as reported by Wibisono and Pusparini 2010[2] and Wibisono *et al*. 2011[3].

1 Franklin, N., Bastoni, S., Siswomartono, D., Manansang, J., & Tilson, R. (1999) Last of the Indonesian tigers: a cause for optimism. In Riding the Tiger: Tiger Conservation in Human-dominated Landscapes (eds J. Seidensticker, S. Christie & P. Jackson), pp. 130–147. Cambridge University Press, UK.

2 O'Brien, T.G., Kinnaird, M.F. & Wibisono, H.T. (2003) Crouching tigers, hidden prey: Sumatran tiger and prey populations in a tropical forest landscape. Animal Conservation, 6, 131–139.

3 Linkie, M., Chapron, G., Martyr, D. J., Holden, J., & Leader-Williams, N. (2006). Assessing the viability of tiger subpopulations in a fragmented landscape. *Journal of Applied Ecology*, *43*(3), 576-586.

4 Wibisono, H. T., Figel, J. J., Arif, S. M., Ario, A., & Lubis, A. H. (2009). Assessing the Sumatran tiger Panthera tigris sumatrae population in Batang Gadis National Park, a new protected area in Indonesia. *Oryx*, *43*(04), 634-638.

5 Sunarto, Kelly, M.J., Klenzendorf, S., Vaughan, M.R., Hutajulu, M.B. and Parakkasi, K., 2013. Threatened predator on the equator: multi-point abundance estimates of the tiger Panthera tigris in central Sumatra. *Oryx*, *47*, 211-220.

6 Dinata, Y. (2008) Assessing the population status and management of tigers in the Batang Hari Landscape, West Sumatra, Indonesia. Master's thesis submitted to the University of Kent.

7 Linkie, M., Haidir, I. A., Nugroho, A., & Dinata, Y. (2008). Conserving tigers Panthera tigris in selectively logged Sumatran forests. *Biological Conservation*, *141*(9), 2410-2415.

8 Dinata, Y. (2010) Protecting Sumatra tigers and rhinos inside and outside of the Batang Hari tropical forests, Sumatra, Indonesia. A report to the U.S. Fish and Wildlife Service Rhinoceros and Tiger Fund (98210-8-G620).

9 Kawanishi, K. & Sunquist, M. (2004) Conservation status of tigers in a primary rainforest of Peninsular Malaysia. Biological Conservation, 120, 329–344.

10 Darmaraj, M.R. 2007. *Tiger monitoring study in Gunung Basor Forest Reserve, Jeli, Kelantan*. Unpublished report. WWF-Malaysia, Petaling Jaya, Malaysia.

11 Rayan, D. M., & Linkie, M. (2015). Conserving tigers in Malaysia: A science-driven approach for eliciting conservation policy change. *Biological Conservation*, *184*, 18-26.

**Supplementary Table 3 | Sources of density estimates and details of data.** To meet population closure assumptions and be consistent with standard sampling procedures, the original and corrected density estimates from Way Kambas[5] come the number of tigers captured over the first six months of the study and not the entire two years. Original grids for two CR studies were unavailable (source codes 1 and 10 below), so these were not included in analyses of buffers bias (*i.e.*, Fig. 1). However, we did correct their effective area and density for use in meta-regression based on the mean correction factor from other CR studies. We did not include densities from studies that used photo capture rates to estimates density (*i.e.*, one from ref. [6]). For Kampar and Peranap forests, where tiger signs were observed but no photos were obtained, we follow Sunatro *et al.* (2013) by assigning them a density equal to one half their lowest SCR density and equivalent standard error (*i.e.*, $0.25 \pm 0.25$ tigers/100km$^2$). For Obrien *et al.* (1999), which is bordered by the Pacific Ocean on one side, we constrained the corrected buffer increment to three sides. For studies lacking information on methods, study design, or CR or SCR results, this was obtained directly from authors.

| | | $\hat{A}$ (W) (km$^2$) | | | $\hat{D}$ (tigers/100km$^2$) and standard error | | | |
|---|---|---|---|---|---|---|---|---|
| | $\hat{N}$ | Half | Full | Fixed | Half | Full | Fixed | Pooled SCR* |
| BBS_Left | 10 | 830 | 1549 | 1545 | 1.16 (0.43) | 0.62 (0.27) | 0.62 (0.22) | 1.16 (*0.53)* |
| BBS_Right | 29 | 511 | 882 | 1545 | 5.77 (2.33) | 3.34 (1.39) | 1.91 (0.82) | 1.16 (*0.53)* |
| Kerinci Seblat | 4 | 917 | 1692 | 1828 | 0.44 (NA) | 0.24 (NA) | 0.22 (0.09) | 0.47 (*0.37)* |
| Gunung Leuser | 6 | 768 | 1416 | 1670 | 0.75 (0.39) | 0.41 (0.24) | 0.34 (0.17) | 0.94 (*0.55)* |
| Kerumutan | 3 | 582 | NA | 884 | 0.52 (0.27) | NA | 0.34 (0.17) | 0.25 (0.18) |
| RimbangBaling | 3 | 354 | NA | 823 | 0.86 (0.50) | NA | 0.36 (0.19) | 0.34 (0.24) |
| TessoNilo 2008 | 8 | 470 | NA | 1269 | 1.7 (0.66) | NA | 0.63 (0.25) | 0.77 (0.32) |
| TessoNilo 2007 | 7 | 617 | NA | 1269 | 1.13 (0.58) | NA | 0.55 (0.22) | 0.87 (0.33) |
| TessoNilo 2005 | 7 | 779 | NA | 1269 | 0.89 (0.38) | NA | 0.55 (0.23) | 0.59 (0.26) |

**Supplementary Table 4 | Effective area sampled and density derived using different CR analytical approaches in this study.** $\hat{N}$ is the estimated number of tigers within the estimated effective area sampled, $\hat{A}$, defined as the minimum convex polygon (MCP) around the CT perimeter plus a buffer. 'Half' denotes the CR approach with $\hat{A}$ estimated using ½ MMDM buffer, 'Full' denotes the CR approach with $\hat{A}$ estimated using MMDM buffer, and 'Fixed' denotes the CR approach with $\hat{A}$ estimated using a buffer derived from the pooled SCR analyses (the 'standardized' or 'corrected' method described in the text and shown to work in the simulations in Supplementary Table 5). Separate densities for forest and non-forest habitats can be calculated in the CR approaches. Capture histories from BBS were analysed separately for each flank. These estimates are less susceptible to underestimating MMDM because we trapped over very large areas, however we still observed that one density value was significantly inflated (BBS_Right) and was corrected. We also include the original (1/2 MMDM), correct (Fixed), and SCR values from the other such study in Sumatra (Kerumutan, Rimbang Baling, and Tesso Nilo; Sunatro *et al.* 2013[4]). Pooled SCR is the mean of forest and non-forest density estimates.

## Supplementary references


1. Joshi, A. R. *et al.* Tracking changes and preventing loss in critical tiger habitat. *Sci. Adv.* **2,** e1501675–e1501675 (2016).
2. Wibisono, H. T. & Pusparini, W. Sumatran tiger ( *Panthera tigris sumatrae* ): A review of conservation status. *Integr. Zool.* **5,** 313–323 (2010).
3. Wibisono, H. T. *et al.* Population Status of a Cryptic Top Predator: An Island-Wide Assessment of Tigers in Sumatran Rainforests. *PLoS ONE* **6,** e25931 (2011).
4. Sunarto *et al.* Threatened predator on the equator: multi-point abundance estimates of the tiger Panthera tigris in central Sumatra. *Oryx* **47,** 211–220 (2013).
5. Franklin, N., Bastoni, S., Siswomartono, D., Manansang, J. & Tilson, R. Last of the Indonesian tigers: a cause for optimism. *Rid. Tiger Tiger Conserv. Hum.-Domin. Landsc.* 130–147 (1999).
6. Maddox, T. *The Conservation of Tigers and Other Wildlife in Oil Palm Plantations: Jambi Province, Sumatra, Indonesia (October 2007)*. (Zoological society of London (ZSL), 2007).
7. Tobler, M. W. & Powell, G. V. Estimating jaguar densities with camera traps: problems with current designs and recommendations for future studies. *Biol. Conserv.* **159,** 109–118 (2013).
8. Royle, J. A., Chandler, R. B., Sollmann, R. & Gardner, B. *Spatial capture-recapture*. (Academic Press, 2013).
