# Supplementary material for: Sumatran tiger survival threatened by deforestation despite increasing densities in parks
Source: Nat Commun. 2017 Dec 5;8:1783. doi: 10.1038/s41467-017-01656-4 (PMC5717059; doi:10.1038/s41467-017-01656-4)
Supplement: Supplementary file 2 — Description of Additional Supplementary Files [file 41467_2017_1656_MOESM2_ESM.pdf]

Description of Additional Supplementary Files

File Name: Supplementary Data 1
Description: Code for simulating data and calculating CR densities using different approaches to estimate $\hat{A}(W)$.
